# Sensitivity to reward and adolescents’ unhealthy snacking and drinking behavior: the role of hedonic eating styles and availability

**DOI:** 10.1186/s12966-016-0341-6

**Published:** 2016-02-09

**Authors:** Nathalie De Cock, Wendy Van Lippevelde, Lien Goossens, Bart De Clercq, Jolien Vangeel, Carl Lachat, Kathleen Beullens, Lieven Huybregts, Leentje Vervoort, Steven Eggermont, Lea Maes, Caroline Braet, Benedicte Deforche, Patrick Kolsteren, John Van Camp

**Affiliations:** Department of Food safety and Food quality, Ghent University, Coupure Links 653, Ghent, Belgium; Department of Public Health, Ghent University, De Pintelaan 185A, Ghent, Belgium; Department of Developmental, Personality and Social Psychology, Ghent University, Henri Dunantlaan 2, Ghent, Belgium; Leuven School for Mass Communication Research, KU Leuven, Parkstraat 45 –box 3603, Leuven, Belgium; Research Foundation-Flanders (FWO), Egmontstraat 5, Brussels, Belgium; Poverty, Health and Nutrition Division International Food Policy Research Institute, 2033 K Street, 20006 Washington, DC USA; Department of Human Biometry and Biomechanics, Faculty of Physical Education and Physical Therapy, Vrije Universiteit Brussel, Brussels, Belgium; Institute of Tropical Medicine, Nationalestraat 155, Antwerp, Belgium

**Keywords:** Sensitivity to reward, Adolescents, Availability, Eeating styles, Eating behavior

## Abstract

**Background:**

Although previous research found a positive association between sensitivity to reward (SR) and adolescents’ unhealthy snacking and drinking behavior, mechanisms explaining these associations remain to be explored. The present study will therefore examine whether the associations between SR and unhealthy snack and/or sugar-sweetened beverage (SSB) intake are mediated by external and/or emotional eating and if this mediation is moderated by availability at home or at school.

**Methods:**

Cross-sectional data on snacking, availability of snacks at home and at school, SR (BAS drive scale) and external and emotional eating (Dutch eating behavior questionnaire) of Flemish adolescents (*n* = 1104, mean age = 14.7 ± 0.8 years; 51 % boys; 18.0 % overweight) in 20 schools spread across Flanders were collected. Moderated mediation analyses were conducted using generalized structural equation modeling in three steps: (1) direct association between SR and unhealthy snack or SSB intake, (2) mediation of either external or emotional eating and (3) interaction of home or school availability and emotional or external eating.

**Results:**

Partial mediation of external eating (a*b = 0.69, *p* < 0.05) and of emotional eating (a*b = 0.92, *p* < 0.01) in the relation between SR and intake of unhealthy snacks was found (step 2). The relation between SR and SSB intake was not mediated by external or emotional eating (step 2). No moderation effects of home or school availability were found (step 3).

**Conclusion:**

Our findings indicate that the association between SR and the consumption of unhealthy snacks is partially explained by external and emotional eating in a population-based sample of adolescents irrespective of the home or school availability of these foods.

## Background

Adolescents often adopt unhealthy eating habits such as a low consumption of dairy products, fruit, vegetables and grains and a high intake of energy-dense snacks and sugar-sweetened beverages (SSBs) [1, 2]. Especially the overconsumption of energy-dense snacks and SSBs in adolescents is on the rise [3–5] and is known to be associated with an excess intake of energy and sugar and a diet failing to meet the national recommendations for adolescence [1, 6–9]. The overconsumption of SSBs has also been linked to overweight and obesity, however for the intake of energy-dense snacks the evidence on its association with obesity is still inconclusive [1, 6]. In Flanders, 27.0 % of adolescents consume sweet snacks every day [10] and respectively 43.8 % and 32.8 % of adolescent boys and girls consume SSBs on a daily basis [11]. Palatable foods, such as energy-dense snacks and SSBs, are found to be particularly rewarding compared to other foods such as fruit [12]. An obesogenic environment, characterized by the omnipresence of palatable foods, is therefore likely to stimulate reward-driven eating at the expense of homeostatic processes [13, 14].

Adolescents’ food choices may be explained by both individual and environmental characteristics [15]. At the individual level, sensitivity to reward (SR) reflects the functional outcomes of the behavioral activation system (BAS) [16]. The reinforcement sensitivity theory explains how BAS is primarily organized by the neurotransmitter dopamine and can be defined as the tendency to engage in motivated approach behavior in the presence or in search of rewarding stimuli such as highly palatable foods [16–18]. SR is higher in adolescence than in childhood or adulthood, SR and rewarding processes might thus play a substantive role in explaining adolescents’ behaviors [19]. However, the level of BAS also differs between individuals, reflected in individual differences in noticing and approaching natural rewarding stimuli [13, 14]. Previous studies have shown that adolescents higher in SR have a higher activation of brain areas implicated in food reward, have higher intakes of energy-dense snack foods and have a greater risk to be overweight [13, 17, 18, 20, 21].

The more recently developed hyper-responsiveness model on SR describes further how a high level of SR might be associated with hedonic eating beyond caloric need and ultimately overweight and obesity [14, 17, 22]. Two different pathways are proposed: eating driven by emotional states (e.g. the emotional eaters) or eating triggered by environmental cues such as the sight and smell of food stimuli (e.g. the external eaters) [14, 17]. To the best of our knowledge, only one study already investigated this hypothesis in adults and found that both overeating (determined by external, emotional and binge eating) and food preferences mediated the positive association between BAS and body mass index (BMI) [17]. External and emotional eating have already been described as stable eating styles in children and adolescents, that could result in habitual patterns of (over)eating [23]. Therefore, these eating styles might explain how a heightened SR fosters palatable and typically unhealthy food and drink intake in adolescents.

At the environmental level, previous research has already shown that the home [24] and school [25] environment are associated with adolescents’ food intake. Access to or availability of palatable snacks and drinks in these environments was associated with higher intakes of these products [24, 25]. The availability of palatable food cues in the environment could trigger individual differences in hedonic eating processes and thereby promote energy-dense snack and SSB intake [13, 17, 20, 26]. Therefore, the environment might interact with SR and its related eating styles, and promote the consumption of energy-dense snacks and SSBs. To the best of our knowledge, only one study found an interaction between SR and fast food exposure on fast food intake in adults [26]. To date no research has focused on the complex interplay between SR, hedonic eating styles and environmental influences in adolescents.

**Fig. 1:**
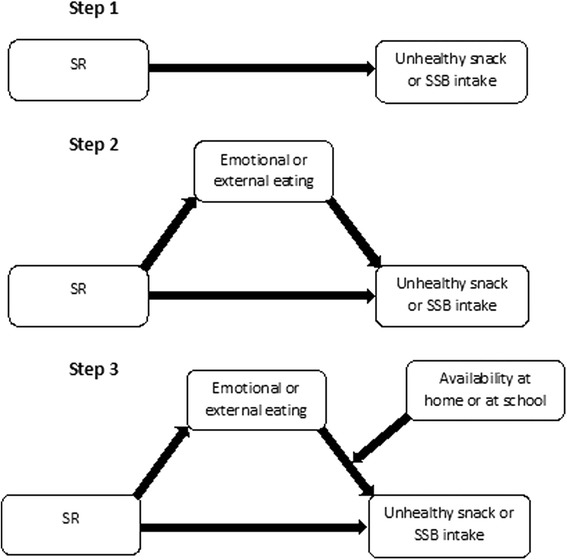
Analytical process

Therefore the present study assessed if the availability of unhealthy snacks or SSBs interacted with elevated levels of hedonic eating styles (external eating and emotional eating) in explaining unhealthy snack and SSB intake. First, the direct association between SR and unhealthy snack and SSB intake was investigated (see Fig. 1). Second, mediation of external or emotional eating on the association of SR with unhealthy snack or SSB intake was examined. Finally, it was assessed if the availability of unhealthy snacks or SSBs at home or at school moderated these mediational pathways.

## Methods

This research was conducted in the context of the REWARD project, a multidisciplinary project that aims to develop reward-based interventions to improve the nutritional status of children and adolescents ^(Vervoort et al., unpublished).^.

### Study procedure and participants

Data were collected from September to December 2013 using a cross-sectional survey in 14- to 16-year-old adolescents from 20 schools in the Flemish region in Belgium. To estimate the variance in SR score with a relative error of 10 % and a 95 % confidence interval (CI), a minimum sample size of 765 adolescents was needed. Further considering a drop out of 15 %, this minimum sample size was set to 900 adolescents. Finally taking into account the design of the study (design effect = 1.2), the final sample size was determined to be 1100 adolescents. The design effect was calculated using a cluster size of 60 students per school and an intra-cluster correlation coefficient of 0.003, estimated from the pilot test of the study in 5 schools not belonging to the study sample. Sample size calculation was executed with the PASS software package (NCSS, Kaysville, UT). To assure a sample size of 1100 adolescents an extra 10 % was sampled. Schools and adolescents were selected using a two-step probability proportional to size sampling procedure. First, schools were randomly selected, stratified by different education networks (public and private), from a list of all secondary schools in Flanders. Second, ± 60 adolescents from each school were randomly selected from a list containing all students in the 3rd and 4th grade. Passive consent was obtained from the parents of the selected adolescents. Eligible adolescents were given two class hours (100 min) on a pre-agreed date to complete the questionnaires in the presence of the research staff in a classroom at their school. The study protocol was approved by the Medical Ethics Committee of the University Hospital Ghent.

### Measures

#### Demographics

Gender was assessed by a one-item question, “are you a boy or a girl?”. Girls were coded as one and boys as zero. Date of birth was asked with an open-ended question, “what is your birthdate?”. Age was then derived by subtracting the date of birth from the date the survey took place. The education type of each adolescent (general/technical/vocational) was obtained from the schools.

### Sensitivity to reward

SR was assessed with the Dutch version of the Carver and White BAS scales for children [27], consisting of three subscales, the BAS reward responsiveness (5 items), the BAS drive (4 items) and the BAS fun seeking subscale (4 items) and a composite scale, the BAS total (all 13 BAS items). All items are answered on a 4-point scale, ranging from totally disagree (1) to totally agree (4). Previous research in children, adolescents and adults has already shown that mainly the BAS drive (DRV) subscale is associated with food intake and eating styles [18, 21, 28] and will therefore be used in this research. Convergent validity and internal consistency of these BAS scales in adolescents have been confirmed in previous studies [29, 30]. In the present sample the Cronbach’s Alpha’s for BAS DRV was 0.81. Scores on the items of BAS DRV subscale were summed and presented as a sum score ranging from 4 until 16.

#### Snack and sugar-sweetened beverage intake

Snack and SSB intake were assessed using a food frequency questionnaire (FFQ). The six categories used were: never or seldom; 1–3 days/month; 1 days/week; 2–4 days/week; 5–6 days/week; every day. Depending on the item, 4–6 portion size categories were provided together with a list of common standard measures as examples. For instance for candy the following portion sizes were given 9 g or less, 10–34 g, 35–59 g, 60–84 g, 85–109 g and 110 g or more, together with the following examples of portions 1 small bag of M&M’s = 45 g and 1 winegum = 4 g. The FFQ probes usual food intake with a reference period of one month. The reliability and validity of this FFQ is reported elsewhere, the FFQ was found valid and reliable on a group level ^(De Cock et al., unpublished)^. In accordance with the definition of Malik and colleagues (2006) of SSBs, the items soft drinks, energy and sport drinks were used to define SSB intake [31]. Unhealthy snacks were defined by classifying the snack items as either healthy or unhealthy using the UK Ofcom nutrient profiling model. This model provides a score that represents the ‘unhealthiness’ of a beverage or food product [32]. Food items that scored more than 4 points were considered to be unhealthy [32]. Following this scoring system, the FFQ snack items crisps, other salty snacks, sausage/cheese rolls and pizza, other fried snacks, fries, hamburgers, cheese or meat cubes, ice-cream, popsicles, breakfast cereals, pudding, sandwiches with sweet or savory spread, mousses, chocolate, candy bars, candy, dry cookies, other cookies, breakfast rolls and pastries were considered to be unhealthy.

The daily intake of each FFQ item was obtained by multiplying the frequency of consumption with the quantity of consumption per week (g) divided by 7. These daily intakes per item were then summed to obtain the daily intakes of unhealthy snacks (g) and SSBs (ml).

#### External and emotional eating

External and emotional eating were measured by means of the Dutch eating behavior questionnaire (DEBQ) [33]. All items were answered on a 5-point scale, ranging from never (1) to very often (5). The DEBQ has been shown to have good factorial validity and dimensional stability and to be suitable for use in an adolescent sample [33, 34]. In the present sample the Cronbach’s Alpha’s were 0.82 and 0.95 for external and emotional eating, respectively.^1^ The average score (ranging from 1 to 5) for both emotional and external eating was calculated by summing the item scores and dividing the sum scores by the number of items^1^.

#### Availability at home

For all FFQ items availability at home was questioned on a 4-point scale ranging from never available (0) to always available (3). The different availability items were recoded into binary variables (0 = never and sometimes and 1 = often and always) and summed to obtain the availability of unhealthy snacks at home (ranging from 0 till 20) and the availability of SSBs at home (ranging from 0 till 3).

#### Availability at school

Availability of unhealthy snacks and SSBs at school was measured using an audit instrument, based on that of the ENERGY project [35]. It comprised the following parts: food and drinks available in the cafeteria/school shop and food and drinks available in the vending machines. Using this instrument a listing was made of all products sold in the cafeteria or in the vending machines. For each school a list was therefore obtained with the number of drinks and snacks sold together with the actual names of all products sold. Based on this document an availability score for SSBs or unhealthy snack items for each school was computed by counting the number of different unhealthy snacks or SSB FFQ items sold at school (either via vending machines or via the school shop) [36].

#### Height and weight

Two trained research assistants measured body height and weight using a standardized protocol [37]. Adolescents were measured without shoes and were allowed to wear light clothing. Body height was measured with a SECA Leicester Portable Stadiometer with an accuracy of 1 mm. Weight was measured with a calibrated electronic scale SECA 861 with an accuracy of 100 g. Age and sex-specific body mass index z-scores (zBMI) were calculated using Flemish 2004 growth reference data [38]. According to the International Obesity Task Force cut-off points, adolescents were classified as either normal weight or overweight [39].

### Statistical analyses

Moderated mediation path analyses were conducted within a multilevel structural equation modelling (MSEM) framework in three steps (see Fig. 1) with three levels of analysis (adolescents within classes within schools). First, the direct association between SR and unhealthy snack and SSB intake was evaluated. In a second step, the mediation pathway of external or emotional eating in the relation between SR and the intake of unhealthy snacks or SSBs was evaluated in two separate models (one for each eating style). Mediation was assessed following Preacher, Zyphur and Zhang [40, 41] for the multilevel 1-1-1 model, using bootstrapped standard errors for the indirect effects. The proportion of the total effect that is mediated, was computed by dividing the indirect effect by the total effect. Step 3 evaluated whether availability of unhealthy snacks or SSBs at school or at home moderated the association between external or emotional eating and unhealthy snack or SSB intake, when evidence of mediation was found in step 2. Moderated mediation was tested following Hayes (2013) [42], including bootstrapped conditional indirect effects when evidence of moderation was found. No evidence of mediation through emotional or external eating was found for the intake of SSBs, therefore moderation mediation was only explored for the intake of unhealthy snacks.

In all steps parameters were mean centered, outliers were removed, unstandardized coefficients and their standard errors were displayed and associations with P-values <0.05 were considered statistically significant. Also in all steps gender, education type and zBMI, were added as covariates, as these were significantly related to the outcomes and we wanted to control for the known influences of demographics (gender and education type) and BMI on food intake. First, the correlation coefficients for zBMI were respectively −0.05 (*p* = 0.09) and −0.08 (*p* < 0.01) for intake of SSBs and unhealthy snacks. Second, the point bi-serial correlations for gender were respectively −0.19 (*p* < 0.001) and −0.20 (*p* < 0.001) for the intake of unhealthy snacks and SSBs respectively. Third and finally, for education type technical the point bi-serial correlations for the intake of unhealthy snacks and SSBs were respectively 0.06 (*p* = 0.06) and 0.02 (*p* = 0.57) and for education type vocational these were respectively 0.11 (*p* < 0.001) and 0.07 (*p* < 0.05). The coefficients shown in the results section are the result of single level generalized SEM (GSEM) as the multilevel models did not provide substantial higher efficiency, based on Akaike’s information criterion (AIC). The multilevel models also did not provide reliable estimates due to the small sample size. The explained variance of the different models was evaluated compared to a null model with no predictors.

A missing value analysis was performed and the missing values, which were in general low (only for zBMI the percentage of missing values was larger than 5 %), were considered to be missing at random. Therefore, no specific adjustment, other than the default missing value procedure of Stata (equationwise deletion), of the analyses was performed.

All analyses were performed using Stata 13.1 (Stata Corporation, Texas, USA).

## Results

### Participants

Of the 1210 selected adolescents, 6 % were absent or not allowed to participate and 3 % returned a questionnaire of unsatisfactory quality (defined as more than 33 % of the questions not completed or straight-lining responses) for further use. The final study sample consisted of 1104 adolescents with a mean age of 14.72 years, 51 % males, 18 % overweight or obese, 46 % following general education, 34 % technical and 20 % vocational. This sample is representative for Flanders regarding gender (51 %, z = 0.11 *p* = 0.92), education type (general 46 %, z = 0.00, *p* = 1.00; technical 32 %, z = 1.42; *p* = 0.16; vocational 22 %, z = 1.60, *p* = 0.11) and the prevalence of overweight or obesity (16 %, z = 1.26, *p* = 0.21) [43, 44].

Other descriptives can be found in Table 1.

**Table 1:** Participant characteristics

*N =* 1104	Mean	SD
Age (y)	14.7	0.8
SR[range 4–16]	9.2	2.9
External eating [range 1–5]	3.0	0.6
Emotional eating [range 1–5]	2.4	0.9
Intake of unhealthy snacks (g/day)	189.9	141.2
Intake of SSBs (ml/day)	234.8	252.4
Availability at home of unhealthy snacks[range 0–20]	8.8	4.9
Availability at home of SSBs[range 0–3]	1.1	0.9
Availability at school of unhealthy snacks[range 0–20]	1.8	2.2
Availability at school of SSBs[range 0–3]	1.2	0.8

### Direct association (step 1)

SR was significantly positively associated to both the intake of unhealthy snacks (b = 7.09, SE = 1.44, *p* < 0.001) and SSBs (b = 8.56, SE = 2.64, *p* < 0.001). 13 % of the total variance in the intake of unhealthy snacks and 9 % of the total variance in the intake of SSBs was explained by SR and the covariates (zBMI, education type and gender), this is an additional 4 % or 1 % respectively compared to the model with only the covariates.

### Mediation analyses (step 2)

**Fig. 2:**
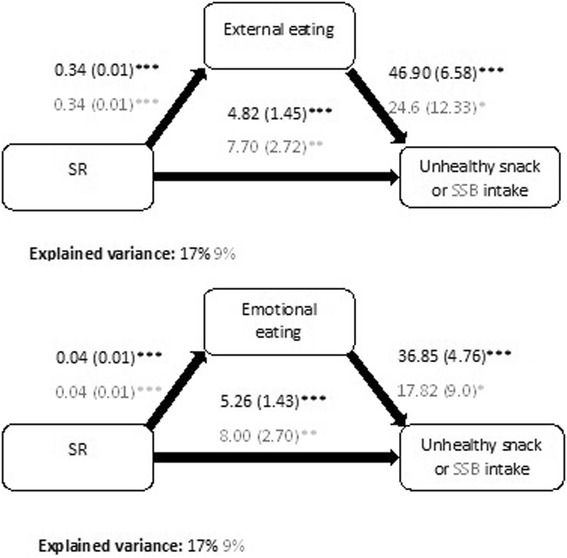
Mediation results. Coefficients are unstandardized and shown in the figure in the format b(SE); *p < 0.05, **p < 0.01 and ***p < 0.001; All analyses were controlled for gender, zBMI and education type

The results of the mediation analyses for both unhealthy snack and SSB intake are shown in Fig. 2. Indirect effects and bootstrapped standard errors are presented in Table 2. Both external eating and emotional eating were partial mediators in the SR- unhealthy snack intake relation. However, neither external nor emotional eating mediated the SR-SSB intake association. Adding the mediational pathway explained an extra 4 % of the variance in unhealthy snack intake for the model with external eating and 4 % for the model with emotional eating. Emotional and external eating respectively mediated 25 % and 23 % of the total effect of SR on unhealthy snack intake.

**Table 2:** Indirect effect and bootstrapped standard errors for the mediation analyses

	Intake of unhealthy snacks				
	Indirect effect (a*b)	Bootstrapped SE	z	p	Normal-based 95 % CI
External	1.60	0.42	3.79	0.000	[0.77, 2.42]
Emotional	1.54	0.43	3.59	0.000	[0.70, 2.38]
	Intake of SSBs				
	Indirect effect (a*b)	Bootstrapped SE	z	p	Normal-based 95 % CI
External	0.84	0.49	1.71	0.088	[−0.12, 1.79]
Emotional	0.75	0.45	1.66	0.098	[−0.13, 1.63]

### Moderated mediation analyses (step 3)

**Fig. 3:**
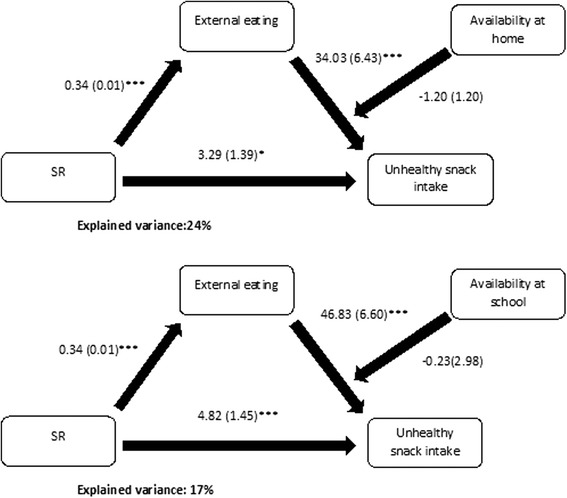
Moderated mediation results with mediator external eating. Coefficients are unstandardized and shown in the figure in the format b(SE); *p < 0.05, **p < 0.01 and ***p < 0.001; All analyses were controlled for gender, zBMI and education type

**Fig. 4:**
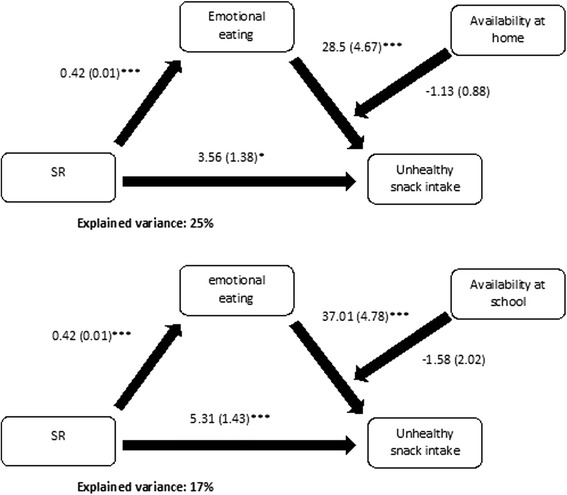
Moderated mediation results with mediator emotional eating. Coefficients are unstandardized and shown in the figure in the format b(SE); *p < 0.05, **p < 0.01 and ***p < 0.001; All analyses were controlled for gender, zBMI and education type

As no evidence of mediation by emotional or external eating on the intake of SSBs was found, moderated mediation was only explored for the intake of unhealthy snacks. Interaction effects of availability at home or at school of unhealthy snacks and external or emotional eating on unhealthy snack intake were non-significant. Coefficients and explained variances are shown in Figs. 3 and 4.

## Discussion

The hyper-responsiveness model depicted how a high SR is associated with hedonic eating (emotional and external eating) and could lead to habitual (over)eating. It was therefore assumed that a high SR would be associated with a higher occurrence of hedonic eating processes, resulting in higher intakes of unhealthy snacks and SSBs. In addition it was expected that the environment in terms of availability would interact with these hedonic eating styles, thereby enhancing their influence on unhealthy snack and SSB intake.

First, the current study found that SR is significantly and positively related to unhealthy snack and SSB intake in adolescents, which is line with previous studies [21]. The present findings therefore provide further evidence for characterizing specifically high SR adolescents as a possible risk group for developing eating and weight problems [17]. The latter is especially important in adolescence, given their overall vulnerability to rewarding processes, as well as to the development of eating problems [19, 45]. However, the explained variance was rather small (unhealthy snacks 9 %, SSBs 8 %). Therefore, and consistent with the multicomponent etiology of overweight/obesity [46] and the biopsychosocial model of eating behaviors [15], it is important to also study other factors such as eating styles, peer influence, parental behaviors and media in relation to unhealthy snacking and drinking behaviors [15].

Second, in line with the hypotheses, two different eating styles were very common in this age group and partially mediated the SR-intake of unhealthy snacks association, namely: external and emotional eating. Davis and colleagues (2004, 2007) previously reported that SR was related to external and emotional eating in adults [14, 17]. The current study extend this observation to the case of adolescents. In addition, the present study also show how a higher SR is associated with the intake of unhealthy snacks through external or emotional eating. However, external eating mediated only 18 % and emotional eating only 23 % of the total effect of SR, therefore other additional mediators should be examined in order to gain more insight into the SR-unhealthy snack intake associations. Examples of other possible mediators are food cravings and food preferences [13, 17]. No mediation by either external or emotional eating of the association between SR and SSB intake was observed. The lack of a mediational pathway through external or emotional eating could be a consequence of the fact that all items of the DEBQ (the scale that was used to measure external and emotional eating) question eating in relation to foods and not drinks [33]. As our results only explained part of the association between SR and unhealthy snack intake and none for the SR-SSB intake association, future research should focus on examining through which other mechanisms SR might influence adolescents’ eating and drinking habits.

Finally, we found no moderation of the association between emotional or external eating with unhealthy snack intake by either availability at home or at school. It thus seems that availability of unhealthy snacks does not interact with external or emotional eating in promoting unhealthy snack intake. This suggests that hedonic eating processes influence adolescents’ snack intake independent of the environment adolescents live in. One other study however did report a significant interaction effect on the intake of fast food in adults when examining the interaction between the environment in terms of fast-food exposure and hedonic factors in terms of SR [26]. The latter discrepancy could be a consequence of the different constructs used to operationalize the environment: exposure implies availability, but not the other way around. Availability just refers to the presence of items in the environment (for example cookies in the highest kitchen cabinet), while exposure also implies access to it (for example the cookies in the highest kitchen cabinet are reachable and visible) [47]. Another possible explanation for this discrepancy when considering availability of unhealthy snacks at school, might be that the variability in these scores was too low to actually observe a moderation. The low variability in availability at school might be due to the design of the study. Data regarding availability was collected at school level (20 schools), the within school variance for these availability measures is therefore zero and the variance is therefore only due to the between-school variance. In order to model the moderation of the school availability of unhealthy snacks it would have been better to use a multilevel model with school as a separate level, but when the whole moderated mediation model was evaluated it was more efficient to stick to a single-level model. The multilevel expansion did not lead to an improvement of BIC or AIC, lead to large computational times and is still considered as a difficult expansion of the normal mediation models [41, 48]. More research is thus needed to investigate how individual hedonic and environmental factors influence adolescents’ food choice alone and/or in combination, particularly regarding the school environment. However it might be if more schools would be sampled and the models could be efficiently estimated as MSEM models that still there would be little or no moderation, as the low variability in availability at schools could also be a consequence of the fact that most schools implemented already similar policies regarding the sales of unhealthy snacks at their school. Therefore it is important to also study the influence of other environments, such as sports or scouting clubs, on adolescents’ snack and drink intake, as these might have a more substantive or a different interaction effect with hedonic eating styles.

This study provides additional insight into how SR, evaluated in terms of BAS DRV, influences unhealthy snack intake in adolescents. External and emotional eating partially mediated the associations between SR and unhealthy snack intake and did not interact with the availability of such items in the environment in promoting unhealthy snack intake. The latter findings emphasize that hedonic eating processes are well-established in adolescents and influence adolescents’ snacking behaviour independent of the environment adolescents live in. Other strengths of this study were the use of a population-based sample, the application of age appropriate instruments, the objective measurements of height and weight and the combination of biopsychological and environmental factors in examining adolescents’ eating behaviors. This study also has some limitations. First, the study design was cross-sectional, so no statements about the causality of the present relations could be made. Second, all collected data except the anthropometrics and snack and SSB availability at school were self-reported and were thus subject to social-desirability bias. We attempted to counter this bias by emphasizing anonymity of the data collection. A third limitation of this study was the length of the survey (±75 min), which could have increased the chance of poor quality answers at the end of the survey e.g. more hurried answers, higher item-nonresponse rates and less variability to items arranged in grids [49]. To avoid this bias, caused by the survey length, three versions of the questionnaire were prepared and administered randomly (except for the demographics, these always came first). A fourth limitation was that total daily energy intake was not measured. This would have increased the burden on the respondents even more, potentially jeopardizing the reporting quality for the key variables. However all analyses were adjusted for bodyweight (zBMI), which according to Jakes and colleagues (2004) has considerable advantages over adjusting for total daily energy intake [50]. A fifth and final limitation was that no measures of pubertal stage or menstrual cycle were taken into account although these could possibly affect energy intake and SR [51, 52].

## Conclusion

First, our findings provide further evidence for characterizing high SR individuals as a vulnerable group for eating and weight problems. Second, our findings also showed that hedonic eating processes may partially explain how a heightened SR leads to unhealthy eating habits and ultimately to overweight and obesity. Finally, we found no evidence that an obesogenic environment, characterized by a high availability of unhealthy palatable foods, enhanced the influence of hedonic eating processes on unhealthy snack intake. For the intake of SSBs no evidence was found that emotional or external eating mediated the SR-SSB intake association. Future research should therefore focus on also exploring other processes that might explain the association between SR and unhealthy eating habits or overweight, and on further examining the possible unique and interactive influences of individual and environmental factors in explaining adolescents’ food choice. Our findings highlight the importance of taking into account individual risk factors, such as sensitivity to reward, in obesity prevention in our current society. As the environment did not interact with SR’s related hedonic eating processes in adolescents, individual strategies will be needed to counter the influence of hedonic eating processes on obesity and overall health. For instance, using positive reinforcement and rewarding strategies to chance eating habits, might be more effective in individuals with high levels of SR^(Vandeweghe et al., unpublished)^. As adolescents are also generally more sensitive to rewarding processes compared to adults and children [19], tailoring based on SR might be an even more promising strategy to prevent obesity and promote healthy food choices in adolescents.

## Abbreviations

AIC: Akaike’s information criterion
BAS: behavioral activation system
BAS DRV: BAS drive
BMI: body mass index
CI: confidence interval
DEBQ: Dutch eating behavior questionnaire
FFQ: food frequency questionnaire
MSEM: multilevel structural equation modelling
GSEM: generalized structural equation modelling
SR: sensitivity to reward
SSBs: sugar-sweetened beverages
zBMI: body mass index z-scores

## Footnotes

1 Item 10 of the external eating subscale, which corresponds to item 33 of the full DEBQ, fell of the questionnaire when printing. This an unfortunate consequence of the magnitude of our study, the long questionnaire and the use of three versions of this questionnaire. However the implications on the results are minimal since 9 other items to measure external eating were present, this tenth item “do you want to eat when you are preparing the food?” is less relevant as a predictor of snack intake as it refers more to the preparation of a meal and this item also showed a low loading factor (0.45) compared to the other items of the external eating scale (0.48–0.66) in a recent validation study of the DEBQ-C by Van Strien et al. [53]. The Crohnbach’s alpha for external eating and the external eating mean score are therefore only based on nine items instead of ten.
